# Revision rate of THA in patients younger than 40 years depends on primary diagnosis – a retrospective analysis with a minimum follow-up of 10 years

**DOI:** 10.1007/s00590-021-02881-w

**Published:** 2021-01-25

**Authors:** Stefan Rahm, Armando Hoch, Timo Tondelli, Johannes Fuchs, Patrick O. Zingg

**Affiliations:** Department of Orthopaedics, Balgrist University Hospital, Forchstrasse 340, 8008 Zurich, Switzerland

**Keywords:** Total hip arthroplasty, Primary diagnosis, Prior surgery, Complication, Revision surgery, Developmental dysplasia of the hip

## Abstract

**Background:**

Treating osteoarthritis in elderly patients with THA is very successful. However, surgeons hesitate to recommend THA in younger patients. The spectrum of etiologies for end stage hip disease in the younger population is diverse and therefore different courses may be assumed. Our objective was to evaluate THA revision rate within a minimum follow-up period of 10 years in young patients and to analyze the difference between different primary diagnoses.

**Methods:**

We included 144 consecutive hips in 127 patients younger than 40 years, who received a primary THA from 01/1996 to 12/2007. Operative reports, clinical and radiographic documentation were reviewed to determine primary diagnosis, prior hip surgery, component specifications and revision surgery. 111 hips in 97 patients were available for outcome analysis with a minimum follow-up of 10 years.

**Results:**

The mean age was 33 years (range 15–40 years) at the time of the index THA, 68 patients were female and 59 were male. Ten years revision rate on the prosthetic components was 13%. The most common primary diagnosis was DDH. DDH was associated with a risk of 17% for requiring a reoperation on the prosthetic components because of mechanical fatigue and therefore, significantly higher than for any other primary diagnosis (*p* = 0.005).

**Conclusion:**

THA in young patients is associated with a high revision rate of 13% in 10 years. 17% of patients with DDH required revision surgery for mechanical fatigue within 10 years, which was significantly higher than for any other primary diagnosis (1.2%, OR 16.8).

## Introduction

Total hip arthroplasty (THA) revision rates are strongly correlated with the patients’ activity and therefore known to be higher in younger patients [1–4]. Furthermore, the type of failure in younger patients differs from an older population and is often of a mechanical cause (e.g., aseptic loosening) [5]. However, implant design as well as surgical approaches and techniques have changed over time and revision rates have decreased [6–8]. While THA may deliver good long-term results even in young patients [9–21], there are also reports about unpredictability of short- to long-term outcome [22–29]. Many studies were designed to identify the best technique or implant design for younger patients, or they focused on a subgroup of patients with a specific primary diagnosis [9–11, 13, 14, 17–21, 30–32]. So far, there is knowledge about the outcome in a mid- to long-term follow-up in patients with juvenile idiopathic arthritis as one of the historically most accepted indications for THA in young patients [30–32]. Recently, more attention is given to the outcome of THA for different non-inflammatory indications in younger patients. Mostly, the influence of prior surgeries or technical aspects on the revision rate is investigated [5, 20, 21, 33]. However, the influence of the primary diagnosis on the risk for revision surgery is still unclear. Our objective was to evaluate THA revision rate within a minimum follow-up period of 10 years in young patients and to analyze the difference between different primary diagnoses.

## Methods

### Study population

**Fig. 1:**
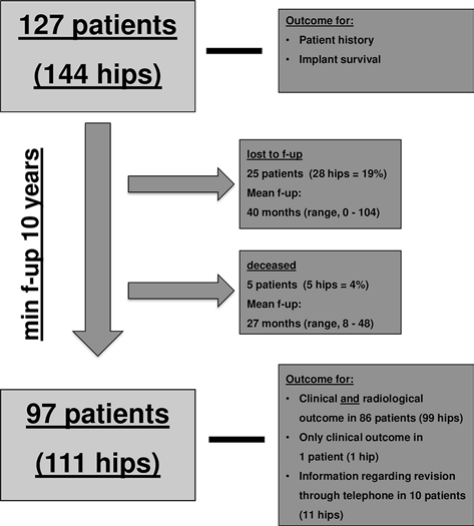
This figure gives an overview over the patient enrollment process with details to the patients lost to follow-up and the outcome parameters

After approval of the study by the responsible ethical review board (KEK Zurich BASEC Nr. 2017–01,616), a retrospective data analysis was conducted. From our institute’s archive, data of all patients under 40 years who received a primary THA in the period from 01/1996 to 12/2007 were retrieved. 127 consecutive patients with 144 THA were identified and invited to participate in the study including a clinical and radiographic follow-up assessment. Of these 127 patients, 30 patients (33 THA) were lost to follow-up within the minimum follow-up period of 10 years: 3 could not be tracked through the local authority, 6 refused to participate, another 4 were lost because they developed a disabling medical condition and were not able to participate in the study, 12 emigrated and 5 deceased (Fig. 1). Finally, 97 patients (111 hips) were evaluated for clinical and radiographic outcome after a minimum follow-up of 10 years. Nevertheless, the 30 patients who were lost, were included in the implant survival analysis according to Kaplan–Meier. At the last follow-up 99 hips (89%) were assessed clinically and radiographically, 1 hip (1%) was assessed only clinically and 11 hips (10%) were assessed through a telephone enquiry.

### Clinical assessment

Our institute’s digital clinical information system was used to search for detailed patient history. Regarding the patients’ history, the primary diagnosis and prior surgeries before implantation of the index THA were assessed. The surgical report was reviewed to determine the surgical approach, the method of fixation of cup and stem and the type of prosthetic components. We analyzed all complications after index THA surgery and the subsequent reoperations, where we differentiated between re-operations with and without revision of the prosthetic components. Major revision surgery was defined as re-operation on the prosthetic components, where we differentiated between re-operations on fixed (cup and stem) and on mobile (head and liner) parts. As patient-reported outcome measures (PROMS) the Western Ontario and McMaster Universities Osteoarthritis Index (WOMAC) [34] and the Harris Hip Score [35] were assessed at the last follow-up after a minimum of 10 years.

### Radiographic assessment

AP pelvis and cross-table lateral view radiographs were available for analysis. Osteolysis around the cup was assessed according to the DeLee and Charnley classification [36], whereas osteolysis around the stem was assessed according to the Gruen classification [37]. The amount of stress-shielding was assessed with the semiquantitative technique developed by Engh [38]. To assess loosening of the stem we quantified a possible subsidence which was determined relevant when at least 2 mm subsidence was present compared to the postoperative radiograph after the index procedure [39, 40]. The presence of spot welds was used as an indicator for stability [41]. Furthermore, heterotopic ossification was quantified according to Brooker [42]. Severe ossification was determined Brooker grade 3 or 4. Eccentric wear was assessed in a qualitative manner and defined as positive when the center of rotation of the head was cranial to the center of rotation of the cup.

### Statistical analysis

Implant survival was calculated using the Kaplan–Meier-estimator. Nonparametric methods were applied due to non-normal distributed data. The effect on outcomes of categorical and continuous variables was analyzed by Fisher’s exact and Kruskal–Wallis test, respectively. In case of statistically significant effects, a post-hoc Wilcoxon rank-sum test was conducted. If not stated otherwise, median and range are reported. The significance level was set at 0.05. Statistical analyses were computed using Stata/IC 15.1 (StataCorp LP, College Station, TX, USA).

## Results

The mean age of all patients was 33 years (range 15–40 years) at the time of the index THA. Sixty-eight patients were female and 59 were male. The distribution of primary diagnosis, perioperative data and component specifications for each hip with a minimum follow-up of 10 years are summarized in Table 1.

**Table 1:** Patient specification

Primary diagnosis		
Developmental dysplasia of the hip (DDH)		29 (25%)
Osteonecrosis of the femoral head (ON)		27 (24%)
Post-traumatic osteoarthritis		21 (19%)
Impingement related osteoarthritis (FAI)		8 (7%)
Ankylosing spondylitis (AS)		5 (5%)
Slipped capital femoral epiphysiolisis (SCFE)		5 (5%)
Legg-calve-perthes-disease (LCPD)		5 (5%)
Rheumatoid arthritis (RA)		4 (4%)
Secondary osteoarthritis after septic arthritis		3 (3%)
Epiphyseal dysplasia		2 (2%)
Hemophilia		1 (1%)
Mukolipidosis		1 (1%)
Perioperative Data		
Prior hip surgery		54 (49%)
Approach	Anterior	27 (24%)
	Trochanter osteotomy	36 (32%)
	Transgluteal	36 (32%)
	Posterior	12 (11%)
Component specifications		
Femoral component	Cemented	73 (66%)
	Cementless	38 (34%)
Femoral head size	22	7 (6%)
	28	100 (90%)
	32	3 (3%)
	36	1 (1%)
Acetabular component	Pressfit	67 (60%)
	Reinforcement ring with cemented inlay	44 (40%)
Bearing	Metal on conventional polyethylene	12 (11%)
	Metal on highly cross-linked polyethylene	79 (71%)
	Metal on metal	12 (11%)
	Ceramic on ceramic	1 (1%)

### Implant survival

**Fig. 2:**
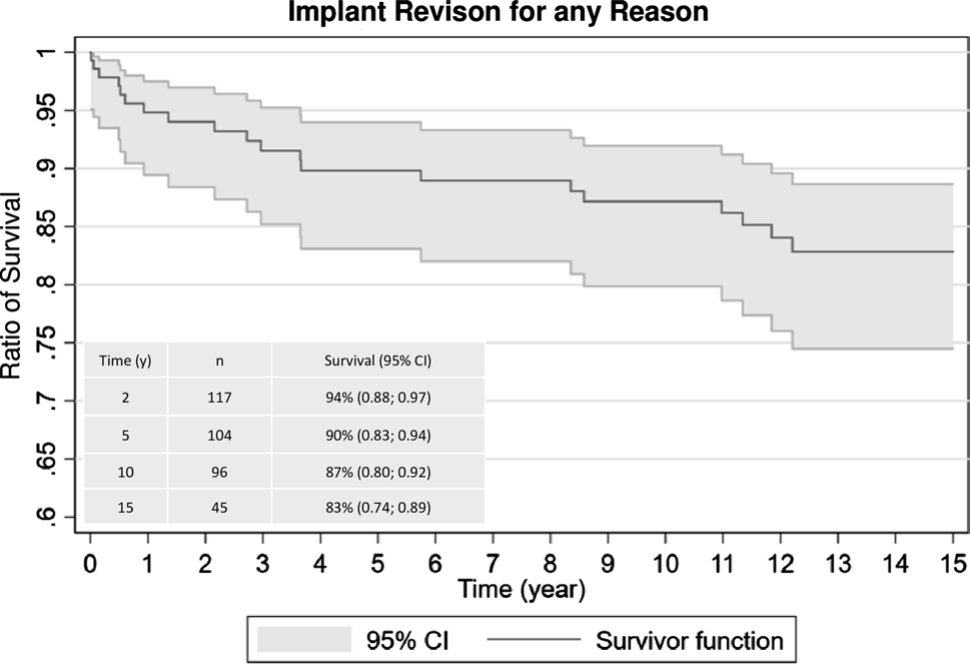
This figure shows the survival rate for the prosthesis within a 15-year follow-up period

The overall implant survival (lack of revision for any reason) was 94% at 2 years (*n* = 117, 95% CI 0.88; 0.97), 90% at 5 years (*n* = 104, 95% CI 0.83; 0.94), 87% at 10 years (*n* = 96, 95% CI 0.80; 0.92) and 83% at 15 years (*n* = 45, CI 0.74; 0.89) (Fig. 2). The 33 hips that were lost to a full 10-year follow-up were tracked for a mean time of 23 months (range 0–104 months). Of the 33 hips that were lost to follow-up before 10 years, two needed a revision on the prosthetic components. Both had a revision of the stem because of a periprosthetic fracture 1 and 18 months after index THA, respectively. In addition, 4 hips needed a minor revision without reoperation on the prosthetic components. These were fixation of a secondarily displaced greater trochanter in one case and hardware removal in 3 cases.

After the follow-up period of 10 years, the re-operation rate on the prosthetic components was 13% (16 hips). In 8% (10 hips) a revision of the stem, and in 10% (12 hips) a revision of the cup was necessary. After the follow-up period of 15 years the revision rate was 17% (20 hips). In 10% (12 hips) a revision of the stem, and in 12% a revision of the cup was necessary.

### Complications

In the 111 hips with a minimum of 10 years follow-up, complications occurred in 46 hips (42%). Of these 46 hips, 4 were treated conservatively and 42 underwent subsequent surgery, whereas 19 underwent minor re-operations without and 23 major re-operations with revision of the prosthetic components. The details are summarized in Table 2. In 8 hips (38%) the cup was revised, in 6 hips (29%) the stem was revised and in 7 hips (33%) both cup and stem were revised.

**Table 2:** Complications

Conservatively treated complications (*n* = 4)		
Fracture^a^		1 (25%)
Temporary nerve palsy		1 (25%)
One-time dislocation		2 (50%)
Surgically treated complications (*n* = 42)		
Reason for minor reoperations		19
Hardware removal		11 (58%)
Internal fixation^b^		3 (16%)
Lengthening of psoas tendon		2 (11%)
Wound revision		1 (5%)
Excision of seroma		1 (5%)
Excision of heterotopic ossification		1 (5%)
Reason for major reoperations		23
Revision of fixed parts		21 (91%)
Mechanical fatigue	Aseptic loosening cup	3
	Aseptic loosening stem	2 (29%)
	Eccentric wear	1
Periprosthetic joint infection		6 (29%)
Irritation of soft tissues		5 (24%)
Breakage of stem		2 (10%)
Periprosthetic fracture		2 (10%)
Revision of mobile parts		2 (9%)
Periprosthetic joint infection		2 (100%)

^a^Vancouver Type AGT, undislocated, ^b^Vancouver Type 1 × AGT, dislocated, 2 × C

Cases of aseptic loosening of the cup, late aseptic loosening of the stem and eccentric wear were merged in order to group the hips showing sequela of mechanical fatigue. No cases of early loosening of the stem were identified. This category of mechanical fatigue related failures was one of the two most frequent reasons for revision on the prosthetic components in 29% (*n* = 6) of all revisions. The implant survival in this group (revision for mechanical fatigue) reached 99% at 2 years (*n* = 111, 95% CI 0.94; 1.00), 98% at 5 years (*n* = 110, 95% CI 0.93; 1.00), 97% at 10 years (*n* = 107, 95% CI 0.92; 0.99) and 95% at 15 years (*n* = 52, CI 0.88; 0.98). The other of the two most frequent reasons for revision on the prosthetic components were periprosthetic joint infection (PJI), which was responsible for 29% (*n* = 6) of all revisions, where both cup and stem were revised. The third most frequent reason was revision of the cup because of irritation of the psoas tendon in cases of anterior uncoverage of the cup or increased tension of the iliotibial band due to non-anatomical reconstruction with increased lateral offset responsible for 24% (*n* = 5) of the revisions (Table 2).

### Subgroup analysis

**Fig. 3:**
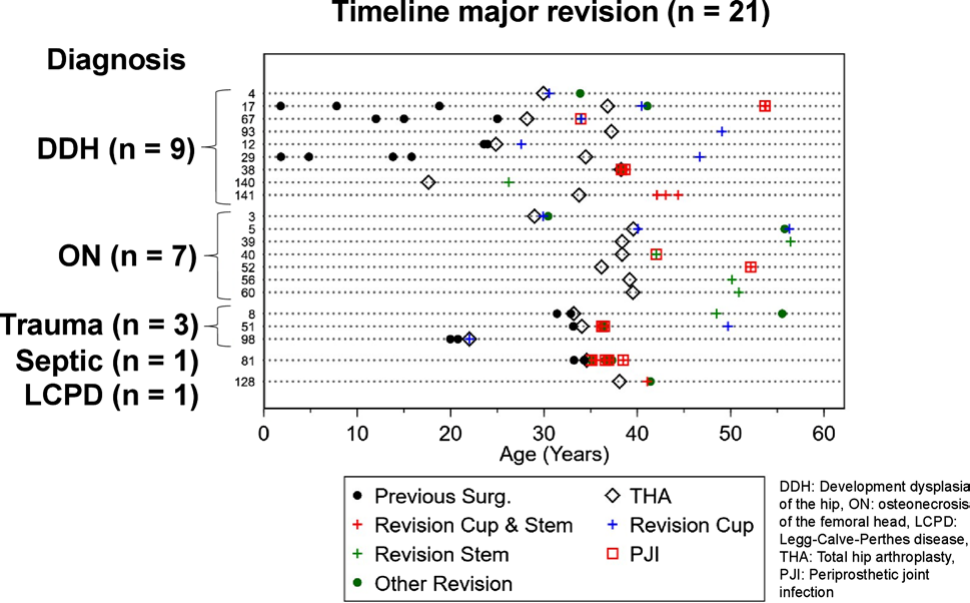
This figure shows the hips, which underwent revisions surgery on the prosthetic components (major revision) with details on primary diagnosis, and the type of previous and revision surgeries

**Fig. 4:**
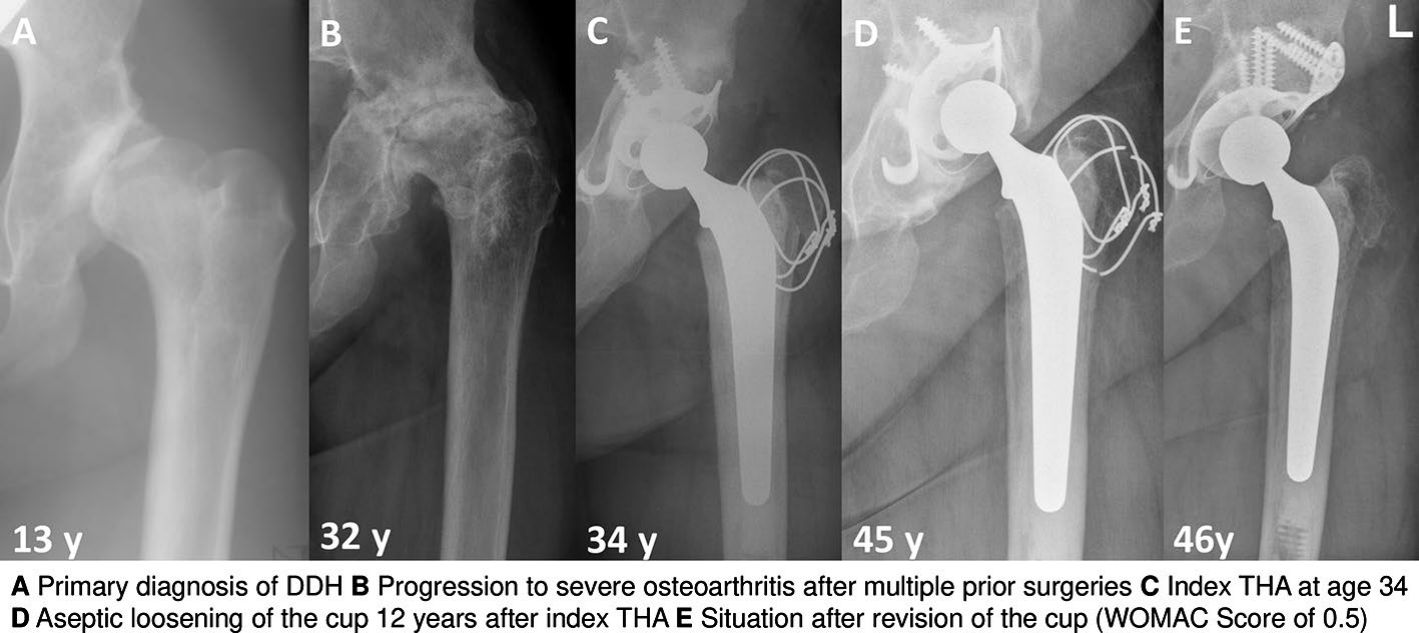
This figure depicts an example case for a patient with DDH, who underwent revision surgery on the prosthetic components due to mechanical fatigue

The primary diagnoses for THA in the patients that needed revision surgery and the type of performed revision surgery are summarized in Table 3. A timeline of the 21 hips with revision of the fixed parts of the prosthetic components is depicted in Fig. 3. Developmental dysplasia of the hips (DDH) as primary diagnosis was associated with a higher re-operation rate on the prosthetic components because of mechanical fatigue compared to the other primary diagnoses (DDH 17.2% vs. other 1.2%; *p* = 0.005, OR 16.8, 95% CI 2.45; ∞). An example case with aseptic loosening of the cup is depicted in Fig. 4. However, DDH was not associated with a higher risk for reoperation on the prosthetic components for any reason (*p* = 0.095). This was due to the fact that the other frequent reasons for reoperation on the prosthetic components (PJI, soft tissue irritation) were equally distributed between the different primary diagnoses.

**Table 3:** Failure patients details

Primary diagnosis	Failure	Months to failure	Revision surgery	WOMAC at final f-up	Bearing	Head Size	Stem	Cup	Bulk bone graft	Anatomy
Developmental dysplasia of the hip (DDH)	Aseptic loosening cup	149	Acetabular revision with allograft and reinforcement ring	0.5	MOXLP	28	Cemented	RR, cemented	Yes	Distorted*
	Aseptic loosening cup	7	Acetabular revision with reinforcement ring	4.6	MOP	28	Pressfit	Pressfit	No	Distorted
	Aseptic loosening cup	44	Acetabular revision with reinforcement ring	2.4	MOM	28	Cemented	RR, cemented	No	Distorted
	Aseptic loosening stem	105	Change of liner and stem	N/A	MOXLP	28	Cemented	Pressfit	No	Distorted
	Eccentric wear	144	Change of cup, liner and head	5.9	COP	28	Pressfit	Pressfit	No	Distorted
	Periprosthetic joint infection	70	Girdlestone, no re-THA	3.3	MOP	22	Cemented	RR, cemented	Yes	Distorted
	Periprosthetic joint infection	1	Girdlestone, re-THA after 5 months	0	COXLP	28	Pressfit	Pressfit	No	Distorted
	Uncoverage of cup, irritation of psoas tendon	33	Change of cup, liner and head	0.2	COXLP	28	Pressfit	Pressfit	No	Distorted
	Periprosthetic fracture	101	Internal fixation, change of cup, liner and stem	N/A	MOXLP	28	Pressfit	Pressfit	No	Distorted
Osteonecrosis of the femoral head (ON)	Aseptic loosening stem	220	Change of liner and stem	0	MOXLP	28	Cemented	Pressfit	No	Regular
	Periprosthetic joint infection	45	Change of liner and stem	0	MOXLP	28	Cemented	Pressfit	No	Regular
	Periprosthetic joint infection (IVDA)	194	Girdlestone, no re-THA	N/A	MOXLP	28	Cemented	Pressfit	No	Regular
	Uncoverage of cup, irritation of psoas tendon	11	Change of cup, liner and head	5.7	MOM	28	Pressfit	Pressfit	No	Regular
	Increased offset, irritation of iliotibial band	6	Change of cup, liner and head	3.2	MOP	28	Pressfit	Pressfit	No	Regular
	Breakage of the stem	134	Change of liner and stem	1.7	MOXLP	28	Pressfit	Pressfit	No	Regular
	Breakage of the stem	138	Change of liner and stem	0	MOXLP	28	Cemented	Pressfit	No	Regular
Post-traumatic osteoarthritis	Periprosthetic joint infection	26	Girdlestone, re-THA after 3 months	4.6	MOXLP	28	Pressfit	RR, cemented	No	Regular
	Uncoverage of cup, irritation of psoas tendon	0	Change of cup, liner and head	0	MOXLP	28	Pressfit	RR, cemented	No	Regular
	Periprosthetic fracture	186	Change of cup, liner and stem	0.6	MOM	28	Pressfit	Pressfit	No	Regular
Secondary osteoarthritis after septic arthritis	Periprosthetic joint infection (IVDA)	6	Girdlestone, no re-THA	N/A	MOXLP	28	Cemented	RR, cemented	No	Regular
Legg-calve-perthes-disease (LCPD)	Uncoverage of cup, irritation of psoas tendon	36	Change of cup, liner and head	0.3	MOXLP	28	Pressfit	Pressfit	No	Distorted

*IVDA* intravenous drug abuse, *THA* total hip arthroplasty, *MOXLP* metal on highly cross-linked polyethylene, *MOP* metal on polyethylene, *MOM* metal on metal, *COP* ceramic on polyethylene, *COXLP* ceramic on highly cross-linked polyethylene, *RR* reinforcement ring, *Distorted: relevant prearthrotic alteration of bony anatomy of the hip (e.g., pathological lateral center edge angle)

**Table 4:** Patients at risk for revision surgery

Eccentric wear		6 (7%)
Relevant osteolysis		5 (5%)
Cup		2
Stem		3
	Late	1
	Distal	2

The history of prior surgery was not associated with a higher risk for reoperation on the prosthetic components (*p* = 0.333). Furthermore, neither the usage of cement for fixation of the stem in primary THA (*p* = 0.073), the head size (*p* = 0.103), the technique of acetabular fixation (*p* = 0.325), nor the material property of the used liner (*p* = 0.476) was associated with a higher risk for re-operation on the prosthetic components.

### PROMS and radiographs at latest follow-up

The median WOMAC at last follow-up was 0.8 points (range 0–5.9), and the HHS was 95 (range 36–100) points for the whole collective. These values were not significantly different from the corresponding values of 0.6 (*p* = 0.95) for the WOMAC and 91.5 (*p*  = 0.55) for the HHS in patients that underwent a reoperation on the prosthetic components.

At the last follow-up radiograph, a subsidence of the stem was present in 3% of the hips. This subsidence occurred within the first year after index THA in all cases and did not progress subsequently. Heterotopic ossifications were seen in 38% of the hips, relevant ossifications were seen in 5% of the hips. Radiographic osteolysis around the stem was present in 20% of the hips, whereas relevant osteolysis in more than 3 of the Gruen zones was present in 3% of the hips (Table 4). Osteolysis around the cup was seen in 5% of the hips, whereas osteolysis of in more than 1 of the DeLee and Charnley zones was present in 2% of the hips. Eccentric wear was present in 7% of the hips, whereas it was significantly more often when a standard polyethylene acetabular liner was used (*p* = 0.026). 9 hips (9%) are all either planned for a revision or the patients are seen on a regular basis in our outpatient clinic due to their radiographic risk profile (e.g., eccentric wear). These hips at risk for revision are listed in Table 4. Two of them showed 2 characteristics to put them at risk: one hip shows eccentric wear which led to late osteolysis around the stem. Another patient shows eccentric wear which led to osteolysis around the cup. The 2 osteolyses around the distal stem are seen in both hips of one patient with ankylosing spondylitis. If these hips were included in the survival analysis, the 10-year survival would be 79% (*n* = 87, 95% CI 0.70; 0.85). Nevertheless, these patients were not all symptomatic.

## Discussion

In this study, we found a rather high rate of 13% of patients requiring a reoperation rate on the prosthetic components after a follow-up period of ten years. A further 9% of our collective are either currently planned to undergo or at a high risk of requiring revision surgery on the prosthetic components in the near future due to wear or osteolysis.

One of the two most frequent reasons for revision surgery on the prosthetic components was failure due to mechanical fatigue. Within this group, developmental dysplasia of the hip was by far the most frequent primary diagnosis and associated with a significantly higher re-operation rate on the prosthetic components (*p* = 0.005). For hips with DDH, the re-operation rate on the prosthetic components due to mechanical fatigue after ten years was 17% compared to 1.2% for hips with other primary diagnoses (OR 16.8). This presumably relates to the fact that these patients have a distorted bony anatomy. The prevalence of prior surgeries before index THA was high in this group. But interestingly, there was no significantly higher risk for a re-operation associated with prior surgery (*p* = 0.333). We believe a major reason for the higher risk for re-operation on the prosthetic components in patients with DDH was the fact that the surgery itself is technically demanding due to the distorted anatomy, such as a shallow acetabulum, a thin anterior wall, a narrow femoral canal and other conspicuous anatomical features requiring deviation from the usual surgical procedure [43]. Interestingly, older patients who underwent THA because of DDH had a similar outcome compared to those with primary OA [44]. This fact may be explained by the lower level of activity in older patients [1–4]. Nonetheless, our collective was young. One the one hand these patients might have had a more severe alteration of the bony anatomy making THA at young age necessary, on the other hand these patients are more active which puts them at higher risk for a revision. Hips with osteonecrosis of the femoral head and post-traumatic OA generally do not have significantly distorted anatomy. This might contribute to the lower risk of requiring a reoperation on the prosthetic components because of mechanical fatigue in these hips.

Overall patient-reported outcome measures reached 0.8 points for the WOMAC and 95 points for the HHS. Therefore, these results are comparable to the general collective of patients undergoing THA [45, 46]. Even patients with DDH after revision surgery on the prosthetic components still showed good outcome measures at the latest follow-up.

In the current literature, successively more attention is paid to outcome measures of THA for different non-inflammatory indications in younger patients. Mostly, the influence of prior surgeries or technical aspects on the outcome is investigated [5, 20, 21, 33]. Kargin et al. evaluated 44 hips in 35 patients younger than 30 years with a mean follow-up of 8 years [20]. Their focus was on the influence of prior hip surgery before index THA on the patient reported outcome and the complication rate. They stated that prior hip surgery did not lead to an inferior outcome. This is in line with the results from our investigation. Swarup et al. included 548 hips in 400 patients younger than 35 years with a mean follow-up of 14 years [21]. Like in our study, the revision free implant survival was 87% after a ten-year follow-up. They were able to show that THA has good short- and mid-term survival in these young patients. Along with younger age within this collective, the type of bearing influenced the outcome. Thus, they reported a ten-year implant survival of 90% in patients ≥ 25 years compared to 82% in patients < 25 years. Ceramic-on-plastic bearings showed a ten-year implant survival of 93% versus 83% in metal-on-plastic bearings. Despite a large collective, they were not able to comment on the influence of non-inflammatory primary diagnoses on the survival rate of the prosthesis.

Few studies investigated the relevance of the primary diagnosis for implant survival in THA [18, 26, 44, 47–49]. Regarding the collective of young patients, even fewer were able to comment on DDH and implant survival [18, 47–49]. Devitt et al. and Kearns et al. both reported about higher revision rates in DDH which is in line with our findings [47, 48]. Kearns et al. reported about an odds ratio of 4.3 regarding the implant revision rate of patients with DDH compared to patients with primary OA, whereas Devitt et al. reported a significantly higher revision rate of patients with DDH compared to patients with primary OA after 20 years. Nevertheless, their collectives were somewhat older with a mean age of 42 and 41 years, respectively, and the nature of the deformity was described as mild, which makes the results difficult to compare with ours. Swarup et al. reported that THA in patients with DDH can have a good outcome [49]. But again, within their collective patients receiving a THA at an age < 25 years are at a significantly higher risk for an implant revision with 23% compared to 10% in patients ≥ 25 years. Hannouche et al. investigated patients who received a THA with a ceramic-on-ceramic bearing at age under 20 years and found a revision rate of 10% after ten years. In the subgroup of DDH, 2 out of 11 patients underwent a revision on the prosthetic components [18].

Our study has several limitations including the retrospective study design and the long period of inclusion with resultant heterogeneity of involved surgeons, used bearings and implants and applied surgical approaches. Additionally, there have been advancements since 1996. However, for a sufficiently large number we included all approaches and all implant types in this study.

## Conclusion

This study confirms that total hip arthroplasty in patients younger than 40 years is associated with a high revision rate of 13% in 10 years. Particularly patients with developmental dysplasia of the hip are at risk for requiring revision surgery on the prosthetic components because of mechanical fatigue, with a revision rate for this indication of 17%, which is significantly higher than for any other primary diagnosis (1.2%, OR 16.8).

## Data Availability

The datasets used and/or analyzed during the current study are available from the corresponding author on reasonable request.
